# Author Correction: Molecular interaction and inhibition of SARS-CoV-2 binding to the ACE2 receptor

**DOI:** 10.1038/s41467-021-23498-x

**Published:** 2021-05-14

**Authors:** Jinsung Yang, Simon J. L. Petitjean, Melanie Koehler, Qingrong Zhang, Andra C. Dumitru, Wenzhang Chen, Sylvie Derclaye, Stéphane P. Vincent, Patrice Soumillion, David Alsteens

**Affiliations:** 1 Louvain Institute of Biomolecular Science and Technology, Université Catholique de Louvain, Louvain-la-Neuve, Belgium; 2 Départment de Chimie, Laboratoire de Chimie Bio-Organique, University of Namur, Namur, Belgium; 3 Walloon Excellence in Life Sciences and Biotechnology (WELBIO), 1300 Wavre, Belgium

**Keywords:** Molecular biophysics, Biophysical chemistry, SARS-CoV-2, Applications of AFM

Correction to: *Nature Communications* 10.1038/s41467-020-18319-6, published online 11 September 2020.

The original version of this Article omitted the following from the Acknowledgements: “Cartoons in Figures 1a–c, 2a, d, 3a, and 4a were created with BioRender.com”.

This has now been corrected in both the PDF and HTML versions of the Article.

