# Supplementary material for: Molecular interaction and inhibition of SARS-CoV-2 binding to the ACE2 receptor
Source: Nat Commun. 2020 Sep 11;11:4541. doi: 10.1038/s41467-020-18319-6 (PMC7486399; doi:10.1038/s41467-020-18319-6)
Supplement: Supplementary file 1 — Supplementary Information [file 41467_2020_18319_MOESM1_ESM.pdf]

Supplementary Information for

Molecular interaction and inhibition of SARS-CoV-2 binding to the ACE2 receptor

Yang *et al*.

This PDF file includes:

Supplementary Figures 1 to 10

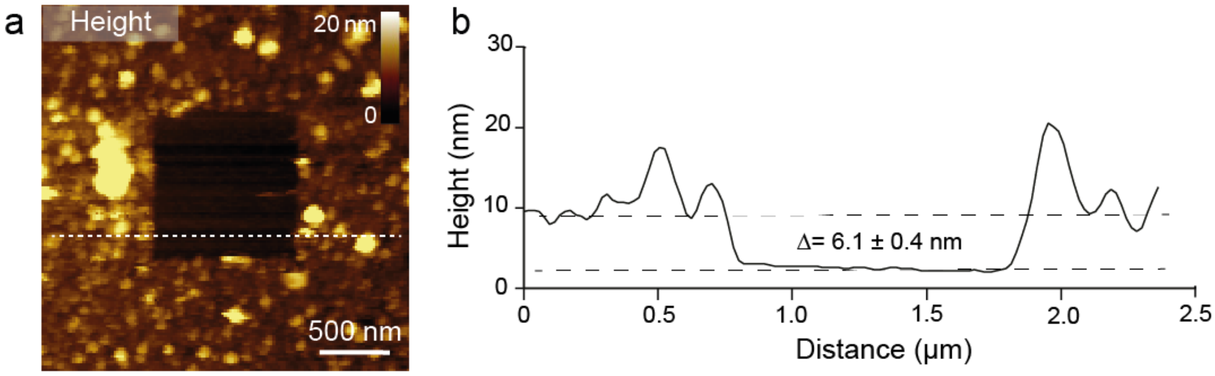

Supplementary Figure 1. Validation of surface immobilization. (**a**) AFM topography image of an ACE2 coated surface after scanning a 1 µm x 1 µm area at high forces (~ 18 nN) to remove the attached biomolecules. (**b**) Cross-section taken along the white dashed line in **a**. The biomolecule-free surface inside the square was ~ 6.1 nm lower in height than the surrounding biomolecule-coated surface, providing an estimate of the thickness of the ACE2 deposited layer. The experiment was repeated 3 times with 3 independent sample preparations.

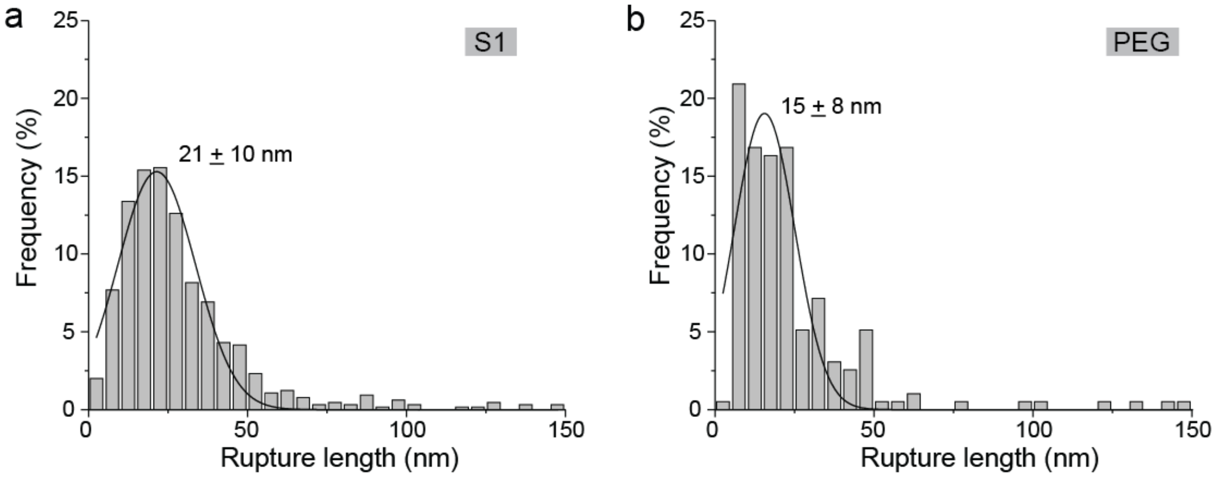

Supplementary Figure 2. Rupture length analysis. Histogram of rupture length for the (**a**) S1-subunit – ACE2 interaction (N = 654 data points) as well as for the (**b**) PEG – ACE2 interaction (N = 196 data points). Multipeak Gaussian fit reveal a maximum of the rupture force at 21 ± 10 nm for the S1-ACE2 interaction and 15 ± 8 nm for the PEG-ACE2 interaction. Source data are provided as a Source Data file.

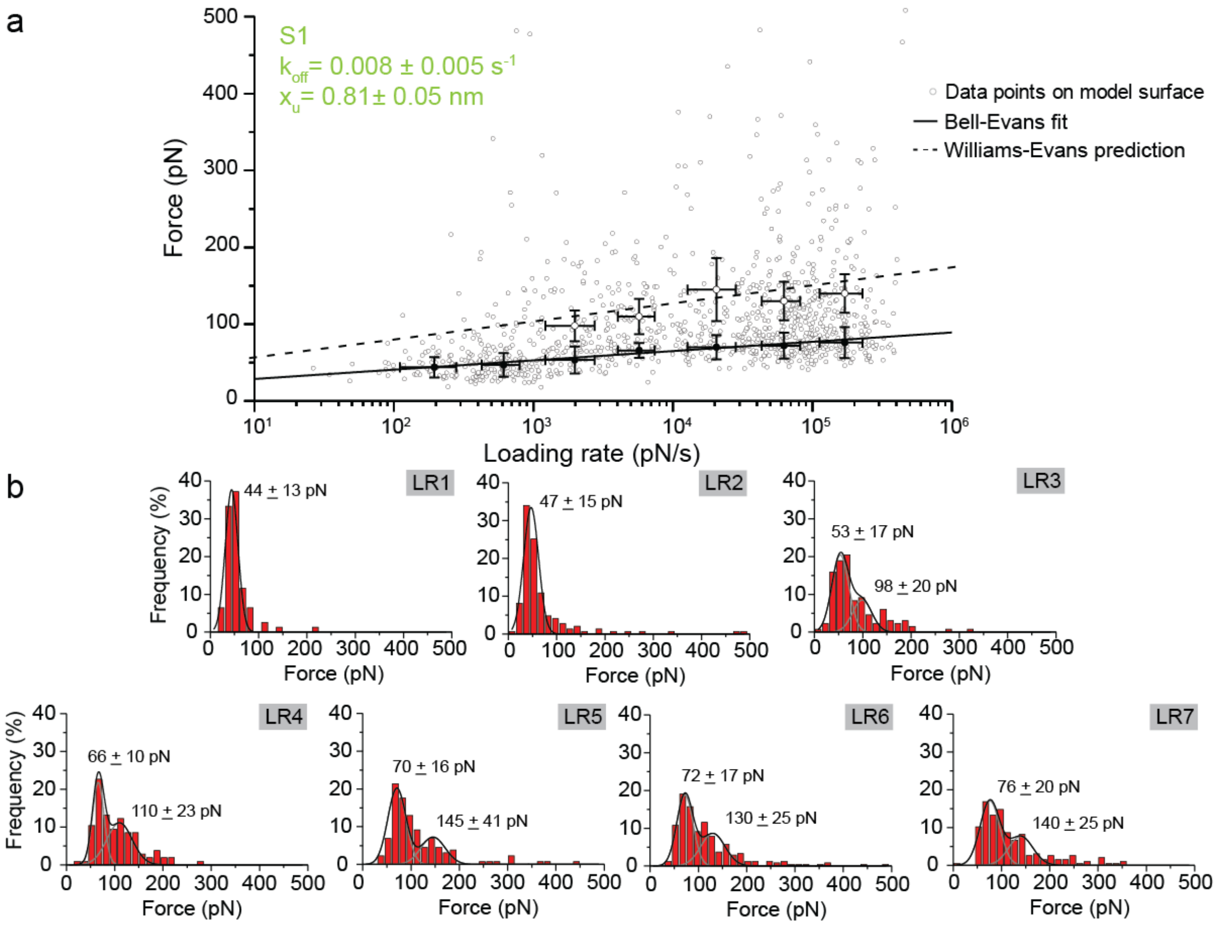

Supplementary Figure 3. Probing S1 binding to ACE2 host receptor on model surface. (**a**) Full-range (0-500 pN) DFS plot showing the distribution of rupture forces measured between S1 and ACE2 coated model surface (grey dots) with average rupture forces determined for seven distinct LR ranges (**b**). Data points corresponding to single interactions are fitted with the Bell-Evans model (black line), whereas the dashed line represent the predicted binding forces for two simultaneous uncorrelated interactions (Williams-Evans model). The error bar indicates s.d. of the mean value. (**b**) Force and LR were extracted from force-distance and force-time curves and sorted in narrow LR ranges (LR1-LR7). The rupture forces for each LR range were plotted as histograms and fitted with multipeak Gaussian fits. N = 1052 from 4 independent experiments.

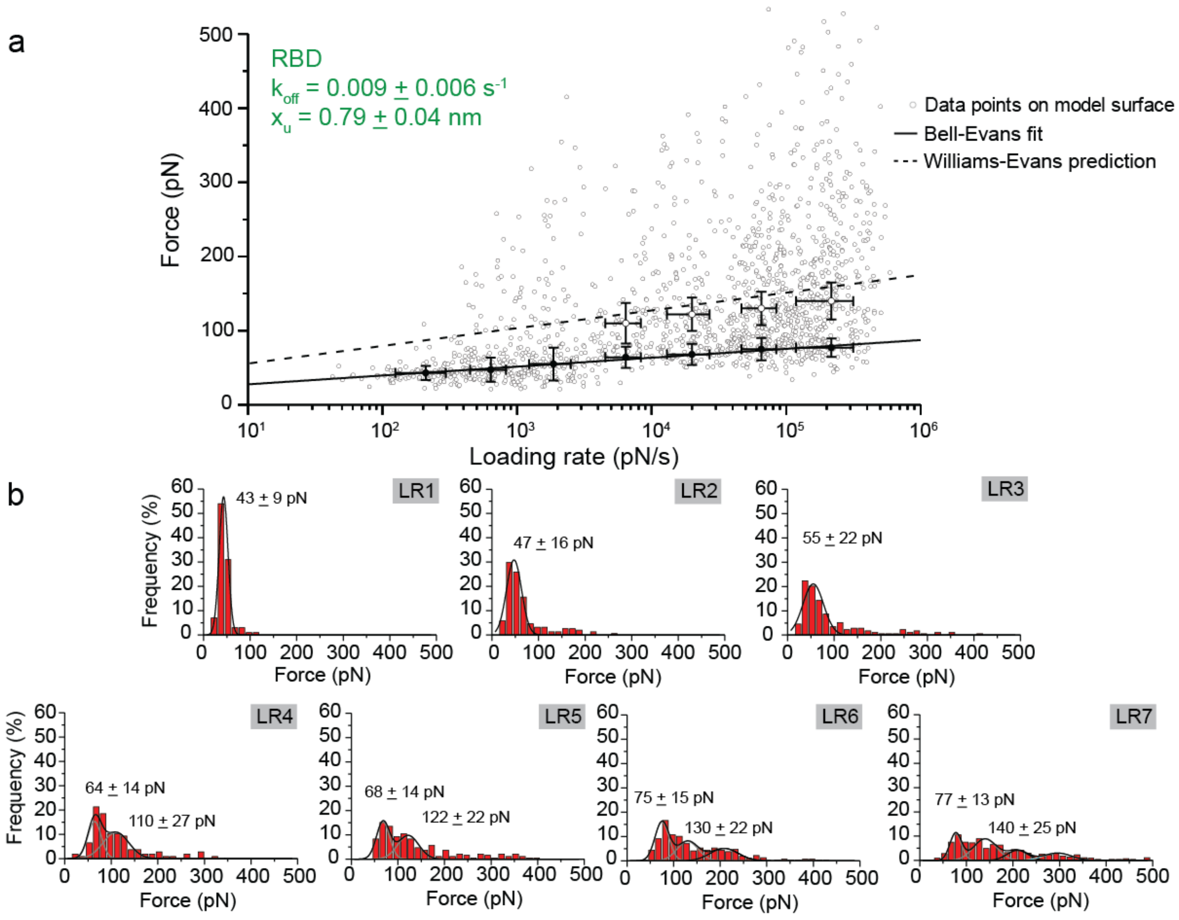

Supplementary Figure 4. Probing RBD binding to ACE2 host receptor on model surface. (a) Full-range (0-500 pN) DFS plot showing the distribution of rupture forces measured between RBD and ACE2 coated model surface (grey dots) with average rupture forces determined for seven distinct LR ranges (**b**). Data points corresponding to single interactions are fitted with the Bell-Evans model (black line), whereas the dashed line represent the predicted binding forces for two simultaneous uncorrelated interactions (Williams-Evans model). The error bar indicates s.d. of the mean value. (**b**) Force and LR were extracted from force-distance and force-time curves and sorted in narrow LR ranges (LR1-LR7). The rupture forces for each LR range were plotted as histograms and fitted with multipeak Gaussian fits. N = 1490 from 4 independent experiments.

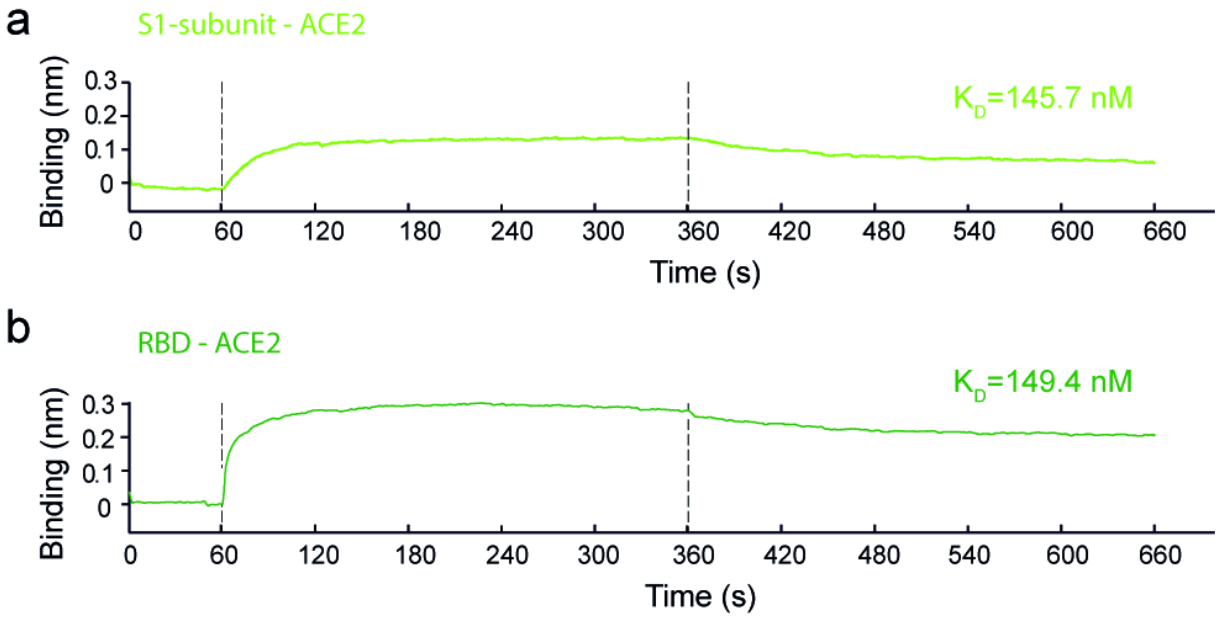

Supplementary Figure 5. Biolayer interferometry data for the binding of S1-subunit and RBD on ACE2 receptors. After a baseline, sensorgram starts with the association (at 60 s) of S1-subunit (**a**) or RBD (**b**) on the ACE2 sensor, followed by the dissociation phase (at 360 s).

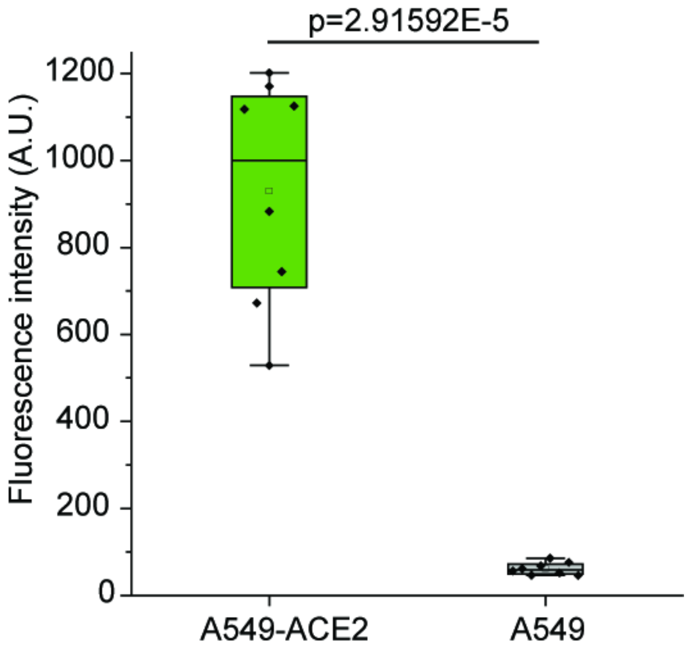

Supplementary Figure 6. Fluorescence intensity of ACE2 transfected (A549-ACE2, green) versus non-transfected (A549, grey) cells. The fluorescence intensity was measured with Zen 3.1 (Zeiss). The same size of area was taken on transfected cells (A549-ACE2) and non-transfected cells (A549) and the average intensity of the area was calculated with Zen 3.1. The statistical analysis was performed with Prism (Graphpad). One data point belongs to the BP from one map acquired at 1 µm/s retraction speed. The square in the box indicates mean, the colored box indicates the 25th and 75th percentile, and the whiskers indicates the highest and the lowest values of the results. The line in the box indicates median. N = 8 cells examined over 3 independent experiments. P-value were determined by two-sample t-test in Origin. Source data are provided as a Source Data file.

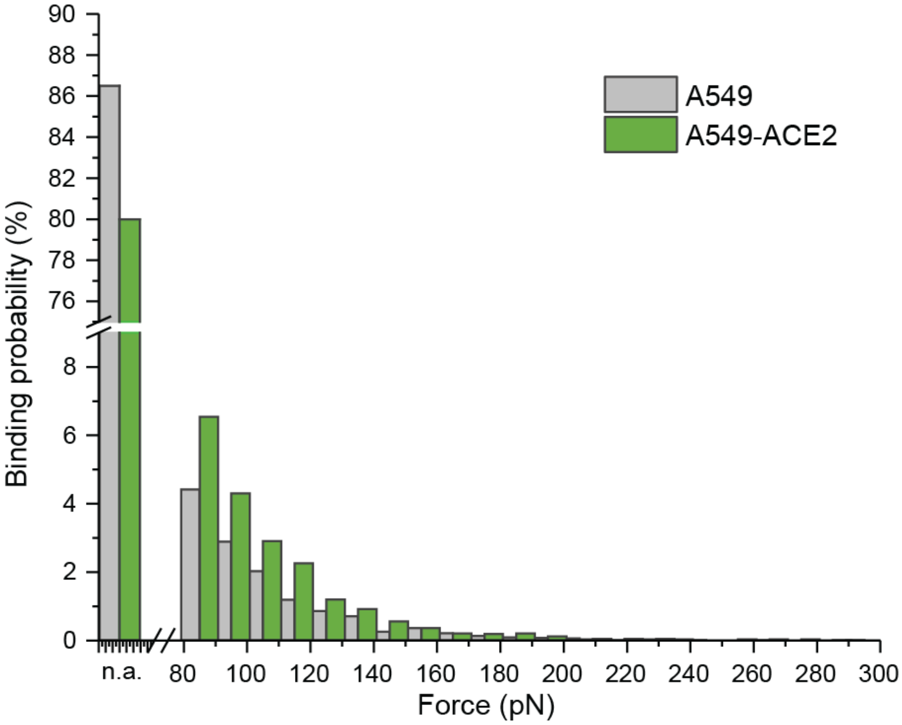

Supplementary Figure 7. Histogram of force distribution extracted on A549 and A549-ACE2 cells (from Fig. 3e). N = 1362 curves on A549-ACE2 cells and N = 891 curves on A549 cells examined over 1 independent experiment. Source data are provided as a Source Data file.

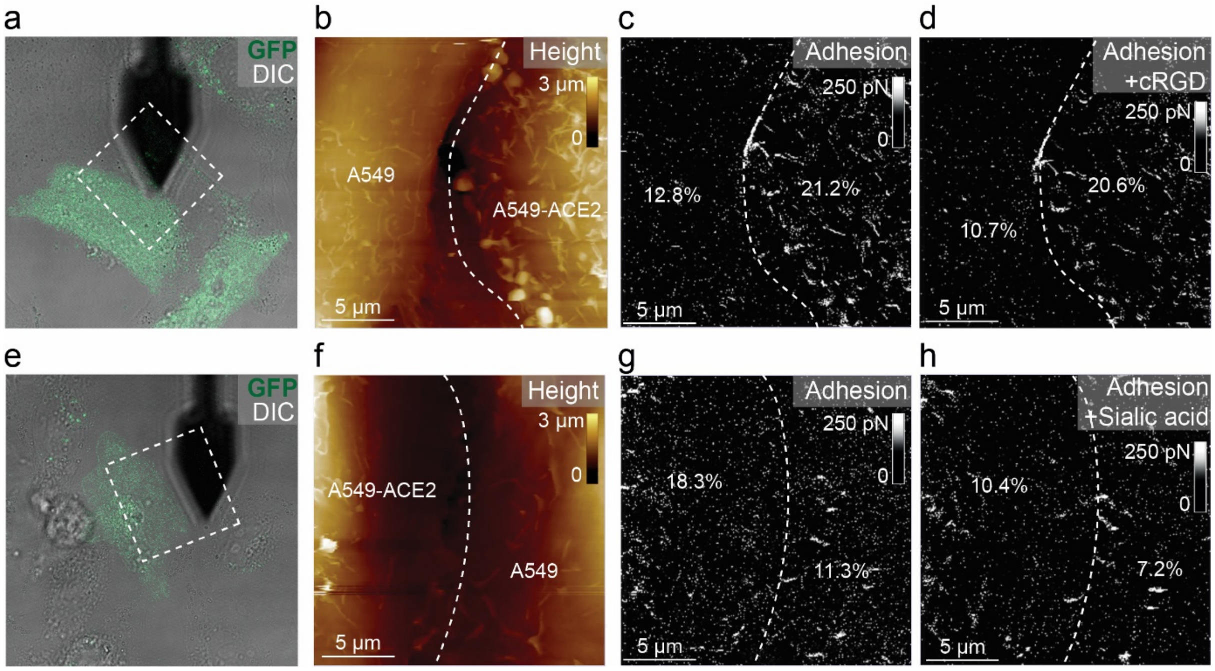

Supplementary Figure 8. Injection experiment to block interactions between living cells and integrins (a-d) or sialic acid (e-h) respectively. (**a**) Overlay of eGFP and DIC images of a mixed culture of A549 and A549-ACE2-eGFP cells. FD-based AFM topography image (**b**) and corresponding adhesion maps (**c,d**) recorded in the specified area in a (scanned with a scan angle) before (**c**) and after (**d**) injection of cRGD to block cell surface exposed integrins. (**e**) Overlay of eGFP and DIC images of a mixed culture of A549 and A549-ACE2-eGFP cells. FD-based AFM topography image (**f**) and corresponding adhesion maps (**g,h**) recorded in the specified area in a (scanned with a scan angle) before (**g**) and after (**h**) injection of sialic acid to block cell surface exposed integrins. The data is representative for at least N = 4 cells from N = 2 independent experiments.

[22-44]	EEQAKTFLDKFNHEAEDLFYQSS
[22-57]	EEQAKTFLDKFNHEAEDLFYQSSLASWNYNTNITEE
[22-44-g 351-357]	EEQAKTFLDKFNHEAEDLFYQSS G LGKGDFR
[351-357]	LGKGDFR

Supplementary Figure 9. Sequences of ACE2-derived peptides.

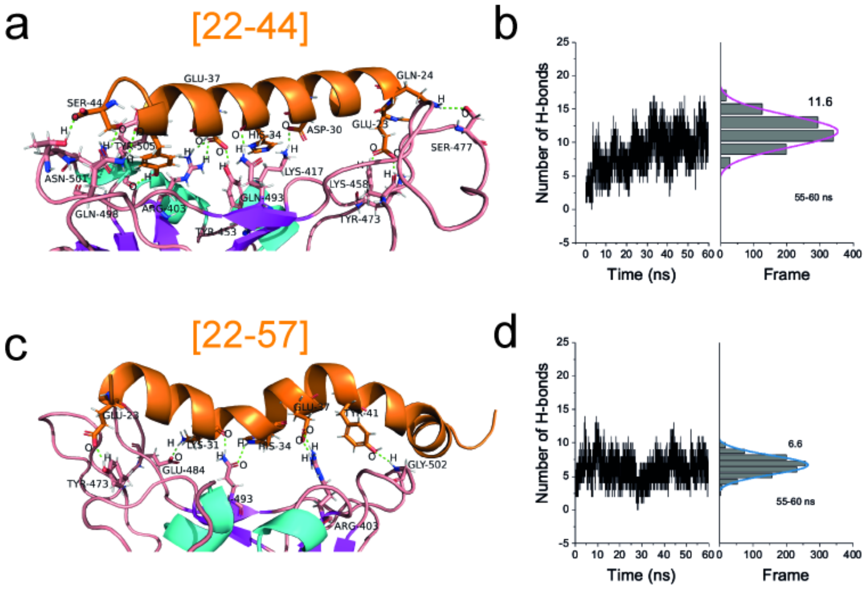

Supplementary Figure 10. Steered Molecular Dynamics (SMD) of ACE2-derived peptides in complex with the RBD. Conformation of peptide 22-44 (**a**) and 22-57 (**c**) in complex with the RBD domain is simulated during 60 ns. During this time frame the number of H-bonds is monitored (**b, d**).
